# Polymers and Solvents Used in Membrane Fabrication: A Review Focusing on Sustainable Membrane Development

**DOI:** 10.3390/membranes11050309

**Published:** 2021-04-23

**Authors:** Xiaobo Dong, David Lu, Tequila A. L. Harris, Isabel C. Escobar

**Affiliations:** 1 Department of Chemical and Materials Engineering, University of Kentucky, Lexington, KY 40506, USA; xiaobo.dong@uky.edu (X.D.); david.lu@uky.edu (D.L.); 2 George W. Woodruff School of Mechanical Engineering, Georgia Institute of Technology, Atlanta, GA 30332, USA; tharris3@gatech.edu

**Keywords:** polymeric membranes, bio-derived solvent, non-solvent induced phase separation, membrane fabrication, scale-up

## Abstract

(1) Different methods have been applied to fabricate polymeric membranes with non-solvent induced phase separation (NIPS) being one of the mostly widely used. In NIPS, a solvent or solvent blend is required to dissolve a polymer or polymer blend. *N*-methyl-2-pyrrolidone (NMP), dimethylacetamide (DMAc), dimethylformamide (DMF) and other petroleum-derived solvents are commonly used to dissolve some petroleum-based polymers. However, these components may have negative impacts on the environment and human health. Therefore, using greener and less toxic components is of great interest for increasing membrane fabrication sustainability. The chemical structure of membranes is not affected by the use of different solvents, polymers, or by the differences in fabrication scale. On the other hand, membrane pore structures and surface roughness can change due to differences in diffusion rates associated with different solvents/co-solvents diffusing into the non-solvent and with differences in evaporation time. (2) Therefore, in this review, solvents and polymers involved in the manufacturing process of membranes are proposed to be replaced by greener/less toxic alternatives. The methods and feasibility of scaling up green polymeric membrane manufacturing are also examined.

## 1. Introduction

Membrane technology has been utilized in liquid and gas separations for decades due to the relative ease in fabrication and operation, high selectivity rates and the absence of sorbent regeneration. In particular, membranes have played an increasingly important role in desalination, water treatment, and food and pharmaceutical industry applications. Membranes can be categorized based on the material of synthesis and they are divided into organic (polymeric) and inorganic membranes. Organic membranes are those made of petroleum-based synthetic polymers, including polysulfone (PS), polyethersulfone (PES), and polyvinylidene fluoride (PVDF), while inorganic membranes include ceramics, carbon molecular sieves, zeolites, amorphous silica, among others. A majority of industrial membranes consist of synthetic or natural polymers. Furthermore, significant amounts of organic solvents are used during membrane fabrication for polymer dissolution [1]; traditional solvents are petroleum-derived and include dimethylformamide (DMF), *N*-methyl-2-pyrrolidone (NMP), and dimethylacetamide (DMAc). However, these component significantly hinder the sustainability of membranes; in particular, traditional solvents used in synthesis and post-synthesis steps can have a negative impact on operational safety and costs, the environment, and human health [2,3,4]. Due to their hazards, solvents require specialized control measures. Therefore, the need for greener, low-toxicity, and more sustainable solvents and polymers has prompted considerable research into the processing of renewable feedstocks to obtain platform molecules and downstream end products. The development and usage of green solvents would allow the global solvent market, which has been on the order of 20 million metric tons and billions of dollars, to align with the United Nations’ Sustainable Development Goals (SDGs) for 2030, as would the integration of green polymers [5,6,7]. Using renewable components derived from biomass, which does not compete with food applications, satisfies both consumer and legislative demands with regards to sustainability.

This review examines the advances in sustainable membrane development and performance. In particular, these advances for polymeric membranes are discussed in terms of phase separation methods, polymers, and solvents due to their prevalent use. In addition to membrane fabrication, evaluating the polymer-solvent interactions, extent of sustainability and scale-up methods are crucial aspects that are examined.

## 2. Membrane Fabrication 

### 2.1. Fabrication Methods

#### 2.1.1. Interfacial Polymerization

**Figure 1:**
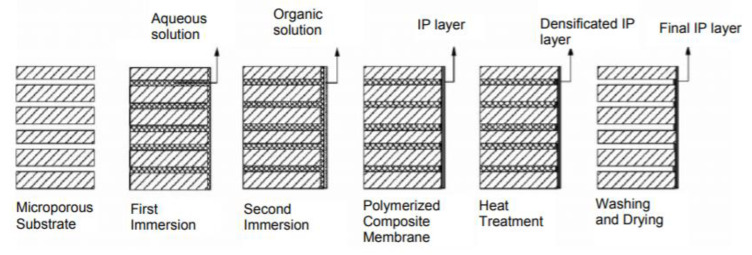
Schematic of interfacial polymerization preparation of TFC membrane. Reprinted with permission from [8], Copyright 2011 IACSIT Publishing.

While phase separations techniques are widely used to cast microfiltration (MF), ultrafiltration (UF) and nanofiltration (NF) membranes, the gold standard method for reverse osmosis (RO) and for thin film composite NF membranes is interfacial polymerization. The development of ultra-thin polyamide membranes through interfacial polymerization was a groundbreaking achievement that set the foundation for modern commercial desalination membranes. Diffusion of amine into an organic solution with acyl chloride or other highly reactive monomers results in formation of a dense polymeric membrane. The resulting membrane is referred to as a thin film composite membrane. As the membrane layer grows, it eventually limits diffusion of amine, thus limiting the active layer to a thickness between 50–200 nm. Among the most common monomers used for nanofiltration membranes are piperazine and trimesoyl chloride (TMC). While TMC is also used in RO membrane, piperazine is less bulky than m-phenyldiamine, which is used in RO membranes and is critical for giving NF membranes selective separations properties. As the interfacial polymerization layer is very thin, interfacial polymerization is also done on UF membranes, so the membrane is formed without defects and has structural reinforcement during pressure-based filtration. A schematic for the general interfacial polymerization process for thin film composite (TFC) membranes is shown in Figure 1 [8].

#### 2.1.2. Phase Separation Methods

Membranes are commonly comprised of polymeric [9,10], ceramic [11,12], stainless steel [13,14], and hybrid materials [15]. From these, polymeric membranes are the most popular due to the high selectivity rates, relative ease of operation and surface feature modifications, and the vast extent of studies [16]. Therefore, this discussion focuses on polymeric membranes and their phase separation-based fabrication methods. Namely, these methods include non-solvent induced phase separation (NIPS), temperature induced phase separation (TIPS), vapor induced phase separation (VIPS) and solvent evaporation induced phase separation (EIPS). Each phase separation method is defined and discussed in terms of several literature studies and compared with respect to advantages and disadvantages. 

**Figure 2:**
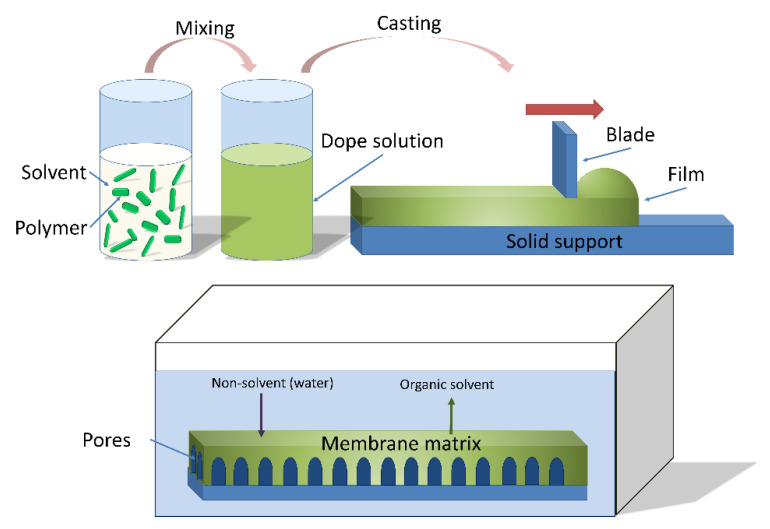
Non-solvent induced phase separation casting process (NIPS). Reprinted from [22], Copyright 2018 MDPI.

Non-solvent induced phase separation (NIPS) is a conventional method to fabricate porous polymeric membranes, as displayed in Figure 2. First, a polymer or polymer mixture is dissolved by at least one solvent to form a homogeneous dope solution; pore formers and other additives that influence the membrane formation may also be included in the solution [17,18]. The dope solution is then cast as a liquid film on a substrate, commonly a glass plate or a polymeric substrate. The liquid film on the substrate is then immersed into a coagulation non-solvent bath, such as water in most instances. Afterwards, phase inversion occurs as the solvent in the film exchanges with the non-solvent [19]. This process results in the formation of an asymmetric polymeric membrane with a dense selective layer and a porous supportive sublayer. These two layers have different functionality; the selective layer provides the separation selectivity for the membranes due to size exclusion or charge, while the porous support layer provides mechanical strength and stability underneath the selective layer [20]. Pagliero et al. [21] used NIPS to prepare polyvinylidene fluoride (PVDF) membranes for membrane distillation and concluded that the principal factor affecting the membrane structure was the rate of crystallization of PVDF during the liquid-liquid de-mixing process.

**Figure 3:**
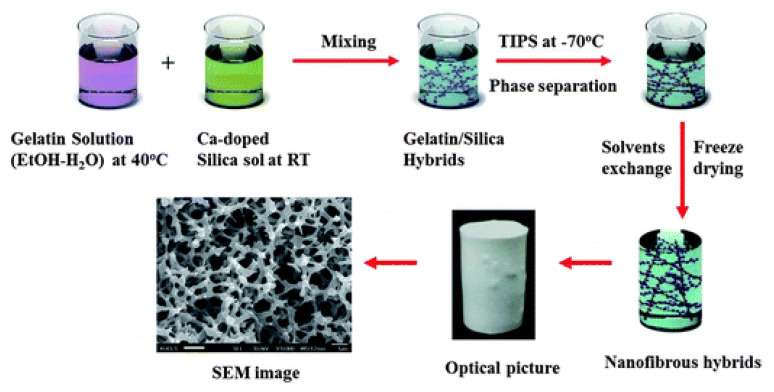
Temperature induced phase separation process (TIPS). Reprinted with permission from [24]. Copyright 2012 Royal Society of Chemistry.

Separately, temperature-induced phase separation (TIPS) is a phase inversion process, as shown in Figure 3, in which a dope solution of polymers and solvents is prepared at a temperature near the melting point of the polymer, and subsequently casted into a film and cooled down to a lower temperature. During the temperature change, phase separation occurs and a solid film forms. Whereas, membranes fabricated using NIPS are usually from ternary systems, TIPS can be used for binary systems, thereby simplifying the process. However, the temperature requirement can limit TIPS as a more energy-intensive fabrication method [23].

M’barki et al. [23] used TIPS along with crosslinking to prepare porous poly(vinyl alcohol) (PVA) membranes. In this study, water was chosen to dissolve PVA to avoid the use of organic solvents. The resulting membranes exhibited connected cellular pores throughout the cross-section of the membranes. However, an open pore structure (larger than 10 μm) was obtained instead of a defect free skin layer due to the use of water as the solvent and higher humidity.

**Figure 4:**
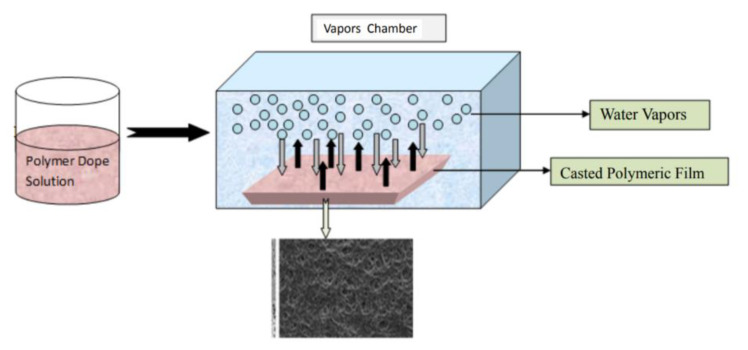
Vapor induced phase separation process (VIPS). Reprinted from [25], Copyright 2018 Journal of Membrane Science and Technology.

Vapor induced phase separation (VIPS) is another method to fabricate porous membranes. As shown in Figure 4, a dope solution is prepared and cast into a liquid film that is then exposed to the atmosphere of the non-solvent vapors, in a vapor chamber. While similar in procedure to NIPS, the phase separation occurs with water vapor being transferred into the film, while the solvent diffuses into the vapor to form a solid membrane film.

Zhao et al. [26] studied the use of VIPS to prepare poly(vinylidene fluoride) (PVDF) porous membranes. The membranes exhibited a cellular structure when the vapor temperature was 65 °C and relative humidity of 70% for 20 min of exposure time. Unlike NIPS, where the dope liquid film is immersed into a non-solvent bath, the dope liquid film was exposed to the vapor phase non-solvent during VIPS, which delays the phase separation process and leads to a cellular membrane structure [26]. It was found that mechanical strength was enhanced, as the cellular structures was bi-continuous [26].

**Figure 5:**
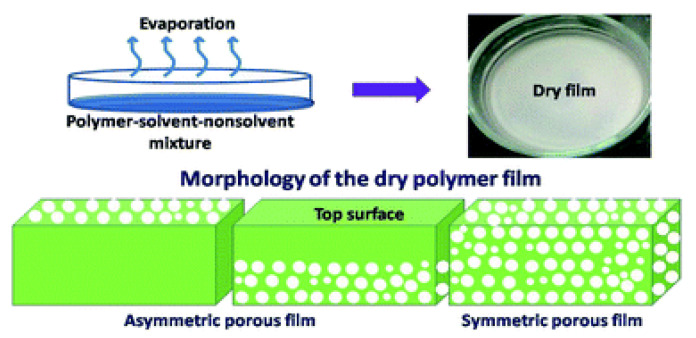
Solvent evaporation induced phase separation (EIPS). Reprinted from [27], Copyright 2019 Royal Society of Chemistry.

In the solvent evaporation induced phase separation (EIPS) method, illustrated in Figure 5, a homogeneous solution is prepared by dissolving a polymer in the mixture of a solvent and a non-solvent, where the solvent has higher volatility than the non-solvent. Through evaporation of the solvent, phase separation occurs and de-mixing of polymer-solvent-non-solvent system occurs, resulting in a porous film. The pore structures can be controlled by changing the composition of polymer-solvent-non-solvent solutions [27].

Samuel et al. [28] investigated the use of EIPS to cast polymethylmethacrylate (PMMA) membranes in tetrahydrofuran (THF) solvent with water as the non-solvent. During the rapid solvent evaporation, condensation of water droplets occurred and formed the porous polymer films. Therefore, water content affected the pore morphology on the membrane surface; the average pore size of the obtained membranes increased along with the water content.

The advantages and disadvantages of the four main phase-separations-based methods are summarized in Table 1. It is important to address that while all these phase separations methods convert a dope solution from liquid to solid, most of the phase separation methods are mass transfer processes, while TIPS alone is based on heat transfer. While other processes have significant differences, it is important to differentiate VIPS and EIPS. First, the mechanisms are different: The non-solvent diffuses into the polymer solution film as vapor in VIPS. In EIPS, the solution film is originally a homogenous polymer/solvent/non-solvent mixture system, and the solvent evaporation promotes phase separation. Furthermore, the driving force of phase separation in VIPS is the diffusion of the non-solvent vapor into the solution film. Whereas, both solvent and non-solvent diffusion from the polymer-solvent-non-solvent liquid film are responsible for phase separation in EIPS [27,29].

From Table 1 and literature studies, it is evident that NIPS can be used to produce different pore morphology, as desired. In fact, NIPS is considered to be the dominant method for fabricating polymeric membranes and has been extensively studied in the literature [23]. Therefore, NIPS was chosen as the principal fabrication method to discuss in this review, thereby minimizing the variables and focus on investigating greener/less toxic polymers and solvents for this method.

### 2.2. NIPS Materials

#### 2.2.1. Polymers

In the fabrication of polymeric membranes, organic solvents have been used in all applications, while polymers have been investigated in different applications. Conventional polymers such as cellulose acetate (CA), polysulfone (PSf), polyethersulfone (PES), polyamide (PA) and polyvinylidene fluoride (PVDF) are studied in different applications, microfiltration (MF), ultrafiltration (UF), nanofiltration (NF), reverse osmosis (RO) and others. CA is a common polymer to make MF. UF and RO membranes; PSf, PES, PVDF are usually used to make UF and MF membranes; PA has been reported to develop membranes used in all the applications mentioned above.

CA is a polymer commonly employed in membrane fabrication and has been extensively researched. CA can be used to prepare microfiltration (MF) [33], ultrafiltration (UF) [34,35], nanofiltration (NF) [36], and reverse osmosis (RO) [37] membranes, and it is usually used as a material for dialysis applications [38]. Unlike other conventional polymers, CA is derived from cellulose, which can be obtained from natural resources and is considered biodegradable. Since cellulose is insoluble, it is processed with acetic anhydride and acetic acid to form CA [23]. However, CA has several disadvantages, such as low chemical resistance, thermal resistance, and mechanical strength [16]. As such, the addition of additives or surface modifications are often needed to improve the properties of CA membranes [35,39,40].

PSf is one of the most prominent polymers used in membrane fabrication. The popularity of PSf is not only due to its commercial availability, but also due to the ease of processing. PSf provides a portfolio of relatively high thermal resistance, chemical resistance and mechanical strength [16].

PES is structurally similar to PSf with suitable chemical and thermal stability [41]. Furthermore, the ether groups within the PES structure allow for easier chemical modifications in comparison to PSf [39,40,41].

Separately, PVDF exhibits high chemical resistance, thermal resistance, and mechanical strength, though it is also notably hydrophobic [42]. The hydrophobicity of PVDF allows for the possibility to be used in membrane distillation [43,44]. Moreover, in order for the membranes to be used in water treatment, surface modification is necessary for increasing the hydrophilicity of membranes [45].

Aside from conventional, mostly petroleum-based polymers, considerable research has been performed on developing and evaluating sustainable polymers. For example, cellulose [46], poly(lactic acid) (PLA) [47], bamboo fiber, chitosan, and others [48,49,50,51,52]. Green polymers have been investigated to minimize the use of petroleum-derived polymers and meet the performance requirements of membranes [53,54,55]. These polymers are derived from natural products, which significantly decrease the carbon footprint of the manufacturing process [56].

Chitosan is a polysaccharide, a polymer derived from the deacetylation of chitin [57,58]. It has numerous advantages, such as being commercially available, environmentally friendly, and having good chemical and thermal stability, biodegradability and mechanical strength. However, finding a solvent that can dissolve chitosan has proven challenging [59,60,61,62]. Acetic acid is commonly used to decrease the pH of a chitosan solution, which increases the solubility of chitosan in the solution [63,64]. However, acetic acid is considered a hazardous solvent [64,65]. Alternatively, Cui et al. [66] used an ionic liquid (IL), 1-ethyl-3-methylimidazolium acetate ([EMIM]AC), in order to dissolve chitosan and prepare membranes. The obtained membranes had a smooth surface without curling and a strong tensile strength of up to 24 MPa, validating that ILs have the potential to be used as alternatives to acetic acid to cast chitosan membranes.

Phuong et al. [48] investigated the use of PLA and bamboo fibers as membrane support materials. PLA is a polyester derived from biomass and is biodegradable. However, the low thermal stability and mechanical strength hindered the use of PLA. Bamboo fiber was then introduced to increase the mechanical stability of the PLA matrix, which was then investigated as a membrane support. In terms of an optimized recipe, the membrane support matrix was found to provide tensile strength comparable to that of a commercial membrane support, as well as higher water permeance.

#### 2.2.2. Solvents

In NIPS, solvents play an essential role in shaping the morphology of membranes and even affecting the properties and performance [16]. During membrane fabrication, large amounts of traditional organic solvents are used [1]. Traditional solvents used in membrane synthesis, including dimethylformamide (DMF), N-methyl-2-pyrrolidone (NMP), dimethylacetamide (DMAc), dimethyl sulfoxide (DMSO), and tetrahydrofuran (THF), have the potential to be hazardous. Traditional petroleum-derived solvents can be highly flammable, irritant, and even have reproductive toxicity [67,68,69]. Due to their hazards, solvents require specialized control measures. In addition to the high toxicity of the solvents used during polymeric membrane fabrication processes [1], energy consumption to remove or recycle solvents from the water is significant [70].

While petroleum-derived solvents have been traditionally used in membrane fabrication, greener/low toxicity solvents are starting to attract attention due to the decreased impacts on human health and the environment from their use [1]. As the world moves towards a more bio-derived manufacturing base, the opportunities for new and bio-derived low hazardous solvents are only expected to increase worldwide. Recently, green solvents have been investigated for membrane fabrication, including methyl lactate, triethylphosphate, ionic liquids, organic carbonates, PolarClean, γ-valerolactone, and others.

##### Methyl Lactate

Methyl lactate is biodegradable, versatile, and has the potential to dissolve CA powders, resulting in a homogeneous dope solution [71]. Gonzalez et al. [71] produced a membrane polymer dope solution using CA and methyl lactate by phase inversion. Prepared using a green process, the resulting membranes were defect-free ultrafiltration membranes. Alqaheem et al. [72] investigated methyl lactate to fabricate polyetherimide (PEI) membranes on the basis that the Hansen solubility parameter indicated methyl lactate had the potential to dissolve PEI, though the experiments subsequently failed to support this notion. The membranes prepared with methyl lactate have exhibited several defects and quality issues, such as inhomogeneity, micro-voids appearing on the surface and varying water permeability [71]. In addition, methyl lactate cannot dissolve a board spectrum of polymers.

##### Triethylphosphate

Triethylphosphate (TEP) has been used as an industrial catalyst in the agricultural industry. Due to its low toxicity, chemical resistance and thermal stability [42], TEP can be considered as a substitute for traditional solvents. Wang et al. [73] prepared polyvinylidene difluoride (PVDF) flat-sheet membranes and a hollow-fiber membrane using TEP as a solvent. Their studies suggested that when TEP was used as a solvent for copolymer blends, a delay in phase separation was observed, and as a result, sponge-like void membranes were formed. Sponge-like membranes resulted in low flux compared to the flux range of finger-like membranes. Tao et al. [74] fabricated PVDF membranes using dimethylformamide (DMF), trimethylphosphate (TMP), hexamethylphosphoramide (HMPA), and TEP by phase inversion and evaluated the resulting membrane performance. It was observed that the membranes prepared using TEP exhibited the lowest flux decline, the highest pure water, and lowest rejection of proteins as compared to other membranes; these trends were mainly attributed to the larger pore size and less compaction of the PVDF/TEP membranes in comparison to the other membranes. The study showed that TEP can be used to prepare PVDF microfiltration membranes. However, the weak mechanical strength of the resulting membranes became a limiting factor to use TEP. Chang et al. [75] also employed TEP to fabricate PVDF hollow fiber membranes for membrane distillation. Without additives, the membranes exhibited a flux of 20 kg/m^2^h at 60 ℃ with an NaCl rejection of 99.99%. However, the mechanical strength of the membrane was compromised; TEP was subsequently introduced in the coagulation bath, which increased the amount of TEP used. Karkhanechi et al. [76] investigated TEP to prepare polyvinylidene difluoride co-chlorotrifluoroethylene (PVDF-co-CTFE) hollow-fiber membranes and compared them to NMP. Based on the analysis of the trinary phase diagram and rheological properties, it was determined that phase separation occurring within the TEP system was easier than within the NMP system, and that the viscosity of the TEP system increased dramatically when water was added into the system. However, it was pointed out that TEP waste may lead to eutrophication in bodies of water, which might stimulate algae growth, resulting in toxic algal blooms and devastation of the habitat of aquatic animals and plants [1,76,77,78].

##### Ionic Liquids

Ionic liquids (ILs) are types of organic salts that consist of an organic cation and a polyatomic inorganic anion. The cation can be imidazolium or pyridinium, while the anion can be a halogen, triflate, or trifluoroborate. Ionic liquids are widely used to replace environmentally toxic organic solvents [71,79,80,81]. Their vapor pressure is often negligible [82]. It should be noted that some ILs (for example, [EMIM][BF_4_] and [BMIM][PF_6_]) have been synthesized with a measurable vapor pressure [83,84]. Moreover, the physical and chemical properties of ILs can be altered by changing the cations and anions to meet requirements for different applications. ILs are non-flammable and generally have high thermal stability [60]. Chichowska-Kopczynska et al. [85] used imidazolium ILs with alkyl fluoride anions in CO_2_ separation and reported that the supported IL membranes were stable and the increase of alkyl chain length would decrease the permeation values of CO_2_. If a trifluoromethanesulfonate anion was used in CO_2_ separation, the solubility of CO_2_ could be lower. Furthermore, supported IL membranes can be used in hollow fiber membrane fabrication. Xing et al. [86] used 1-butyl-3-methylimidazolium thiocyanate ([BMIM][SCN]) to prepare flat-sheet and hollow-fiber CA membranes and compared them to membranes prepared using traditional NMP and acetone solvents. The membranes prepared with ILs exhibited a denser structure; it was also reported that ILs could be recycled and reused to fabricate membranes. Xing et al. [87] used 1-ethyl-3-methylimidazolium thiocyanate ([EMIM]SCN) and 1-ethyl-3-methylimidazolium acetate ([EMIM]OAc) to fabricate CA hollow-fiber membranes. [EMIM]OAc interacted with CA more than [EMIM]SCN, while the CA/[EMIM]OAc dope solution presenting a more highly entangled network than the CA/[EMIM]SCN dope solution. Therefore, the CA/[EMIM]OAc system was more practical for fabricating CA membranes. Colburn et al. [88] also investigated [EMIM]OAc to fabricate cellulose/graphene quantum dot (GQD) membranes. Since cellulose is difficult to dissolve in common solvents, dissolution of cellulose in an IL was evaluated in this study. Within the IL, GQDs were incorporated homogeneously into the cellulose membranes, which improved the membranes performance in regard to photoactivity and sensing. However, the viscosity of dope solutions significantly increased, which has the potential to lead to deficits on the surface of the membranes during the phase inversion process.

It is important to note that the synthesis of ILs is neither clean nor energy-efficient; hence, the cost of using ILs could be high [79]. The toxicities of ILs may vary significantly across organisms and tropic levels [79,89,90,91,92]. Furthermore, the biodegradability of ILs is slow [89]. Considering these perspectives, ILs may be considered adequate, though they may not be considered as ideal “green” substitutes for conventional solvents.

##### Organic Carbonates

Organic carbonates are classified as esters of carbonic acid and are commonly used as solvents, including propylene carbonate, glycerol 1,2-carbonate, and butylene carbonate. These solvents generally have green properties, namely low toxicity, biodegradability, and being synthesized in supercritical CO_2_ [93]. Despite usage in other applications, studies on organic carbonates as green solvents for membrane fabrication are currently limited. Recently, Rasool et al. [93] used NIPS to prepare NF membranes from 15% cellulose triacetate (CTA) in dimethyl carbonate (DMC)/NMP and 15% PES in PC/NMP solvent mixtures with rejections exceeding 90% and permeances of 17.2 LMH/bar and 10.8 LMH/bar, respectively; mixing the green solvents with NMP in a 2:1 ratio aided in the dissolution of the polymers and decreased the total volume of hazardous solvent used [93].

##### Rhodiasolv^®^ PolarClean

**Figure 6:**
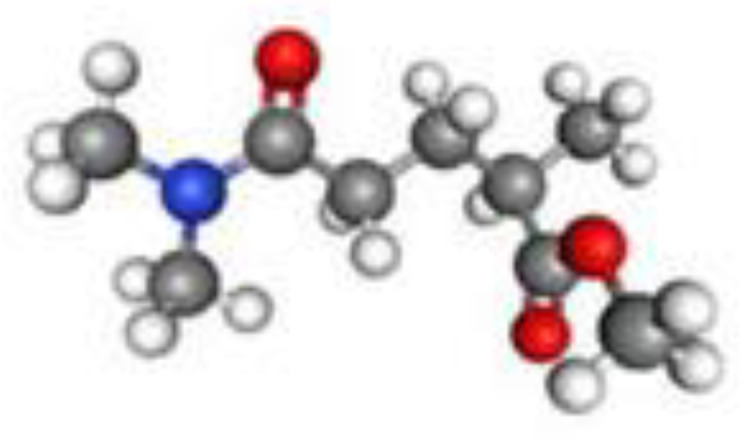
Chemical structure of Rhodiasolv^®^ PolarClean (Methyl-5-(Dimethylamino)-2-Methyl-5-Oxopentanoate).

PolarClean is a water-soluble, eco-friendly, and biodegradable polar solvent, as shown in Figure 6, with no reported health hazards when used for casting PVDF membranes [94,95]. It is a green solvent commercialized by Solvay Novecare and is derived from the valorization of 2-methylglutaronitrile (MGN), which is a byproduct from the synthesis of Nylon 6,6 [96,97]. As such, the production of PolarClean can reduce the carbon footprint and minimize the environmental impact [96]. Hassankiadeh et al. [94] used PolarClean to fabricate PVDF hollow-fiber membranes via TIPS. However, the rate of PolarClean outflow from the PVDF/PolarClean system was observed to be higher than the rate of water inflow; this difference resulted in dense hollow-fiber membranes with low-water permeability. Due to the high miscibility of PolarClean with water, phase separation can be affected by both temperature changes and the diffusion of water and solvent during membrane fabrication, thus, indicating the presence of a NIPS effect during the TIPS process. Jung et al. [95] investigated the combined TIPS-NIPS (N-TIPS) effect on the membrane surface during the fabrication process, along with the kinetics of the membrane formation process. By increasing the coagulation bath temperature and polymer concentration, the phase separation and membrane morphology were primarily influenced by TIPS. In addition, over-dense top layers were also reported and required a pore-former, such as Pluronic F-127, to improve water permeability at the expense of mechanical properties [95].

##### Gamma-Valerolactone

**Figure 7:**
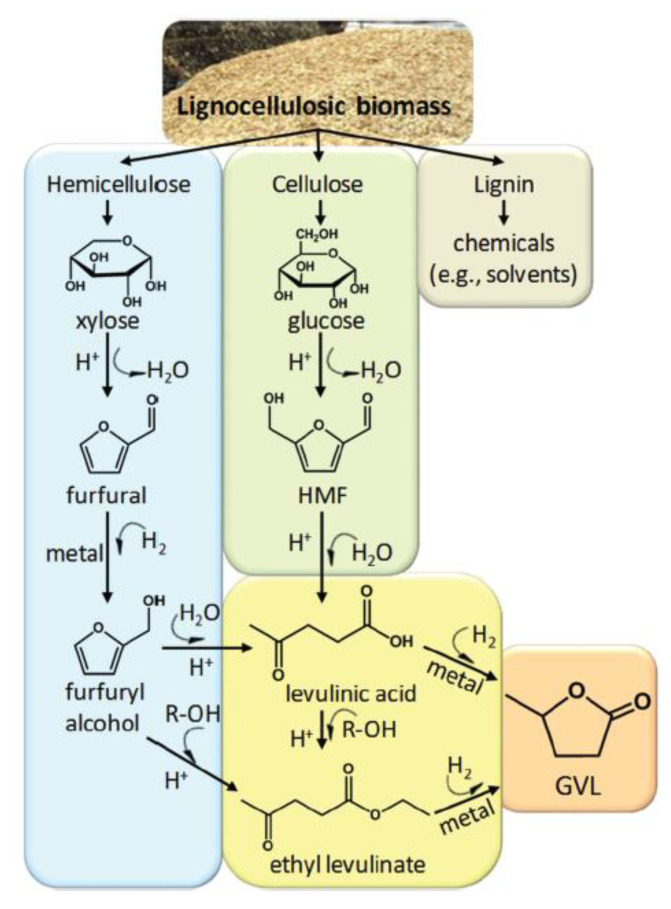
Lignocellulosic biomass and reaction pathways to produce GVL. Reprinted with permission from [98]. Copyright 2013 Royal Society of Chemistry.

Gamma-valerolactone (GVL) is a 5-carbon cyclic ester with 5 atoms in the ring. It is water-soluble and can be bio-derived from lignocellulosic biomass, specifically from hemicellulose and cellulose, according to the process shown in Figure 7 [98]. Briefly, hemicellulose is converted to furfural and furfural alcohol as intermediates by acid hydrolysis; the furfural alcohol is then esterified with ethanol to produce ethyl levulinate [98,99,100]. Cellulose is converted to hydroxymethylfurfural (HMF) as an intermediate and then converted to levulinic acid also through acid hydrolysis [99,100]. Both ethyl levulinate and levulinic acid are hydrogenated to GVL [98]. Rasool et al. [101] prepared membranes using GVL using a variety of different polymers, most notably CA and cellulose triacetate (CTA). Specifically, 15% CA/GVL and 10% CTA/GVL dopes were used to cast nanofiltration (NF) membranes that rejected 90% Rhodamine B at permeances of 1.8 Lm^−2^ h^−1^ bar^−1^ (LMH/bar), and 11.7 LMH/bar, respectively.

##### PolarClean and GVL as Co-Solvents

**Figure 8:**
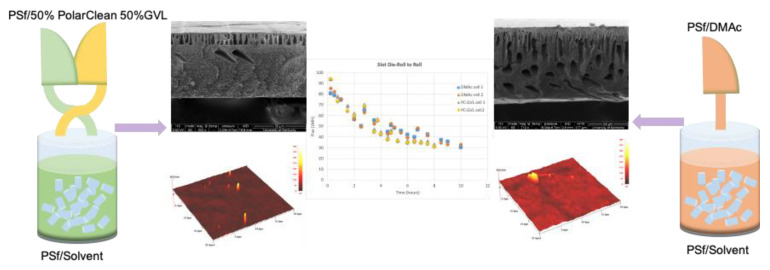
Cross-sectional morphologies, atomic force microscopy imaging to show roughness, and comparison of flux decline for membranes made from dopes that used the PolarClean and GVL as co-solvents to those made using the petroleum-derived solvent.

Dong et al. used PolarClean and GVL to dissolve PSf to fabricate ultrafiltration membranes both as sole solvents and as co-solvents. When PolarClean was used as a sole solvent, it produced membranes with sponge-like pore structures that were different from the finger-like structures, observed when DMAc was used to cast PSf membranes [22]. Furthermore, the PSf/PolarClean pore structure collapsed upon backwashing, which made the water flux after backwashing decrease; these were considered not optimal. Conversely, GVL alone was found not to be suitable to fabricate PSf membranes because the dope formed gel-like films instead of solid films during non-solvent-induced phase separation (NIPS) with water as the non-solvent [102], so a viable membrane was not produced. On the other hand, under equal amounts of PolarClean and GVL as a co-solvent mixture, it was observed that membranes had similar structural, morphological and operational properties compared to membranes made using the petroleum-derived and toxic solvent, DMAc [103], as shown in Figure 8.

Given the green solvents in this review, it is evident that numerous alternatives exist for potentially replacing traditional solvents. Furthermore, the derivation of green solvents from various sources and their potential usage as single or co-solvent mixtures support their versatility for membrane applications. As more green solvents are developed and evaluated, the prospect of replacing traditional solvents and overcoming their limitations is becoming more feasible, thereby reducing the hazards and environmental impacts of membrane fabrication. On the other hand, there are drawbacks and concerns towards the use of green solvents. For example, the prices of many green solvents are higher than those of petroleum-derived solvents. The cost of distillation of these different green solvents should be investigated and compared to determine the economic feasibility of solvent recovery. Last, these solvents are biodegradable so that they may cause eutrophication of receiving waters.

### 2.3. Influencing Factors on Membrane Morphology

The morphology of polymeric membranes is dependent on numerous factors, including the polymer(s) and solvent(s) used in the dope solution. Regardless of the component selection, several factors related to NIPS can influence the morphology, namely evaporation time, casting thickness, demixing path and diffusion rate of solvent/non-solvent.

One of the advantages to using phase separation methods is the number of adjustable fabrication parameters that can factor into the membrane morphology and performance. Aside from the dope solution composition, one external parameter that is often adjusted during phase separation is the amount of time the casted solution film is exposed to air before non-solvent immersion, known as evaporation time or an evaporation step. The addition of evaporation time is particularly useful for systems with a volatile solvent/co-solvent as the volatile solvent/co-solvent is selectively reduced, the polymer concentration in the top “skin” layer increases and acts as a resistance barrier between the non-solvent bath and bulk membrane layers during non-solvent immersion. This increased resistance limits the diffusion of non-solvent into the membrane and delays the de-mixing process [104].

The influence of evaporation time on polymeric membrane morphology has been examined in several studies. Holda et al. [105] investigated the relationship between evaporation time and membrane morphology in PSf solvent resistant nanofiltration (SRNF) membranes. SEM images of membrane cross-sections indicated that the amount of microvoids found underneath the top skin layer decreased as evaporation time increased and completely disappeared once evaporation time surpassed 120 s. Moreover, the formation of the resistance barrier at the top surface resulted in a denser skin layer with increasing evaporation time [105]. Hendrix et al. [106] reported similar observations in the fabrication of poly(ether ether ketone) (PEEK) membranes, in which membranes fabricated at longer evaporation times exhibited fewer microvoids due to the increased polymer concentration decreasing water diffusion during NIPS. As such, evaporation time may serve as a tool for densifying membranes [106].

During the immersion of the polymer film in the non-solvent coagulation bath, solvent diffuses out of the film while the polymer film solidifies. As a result, the volume and thickness of the film decrease, even leading to the formation of membranes with half the thickness of the initially casted polymer film in cases [106]. Manipulation of the casting thickness has influenced this process and produced different membrane morphologies. PEEK membranes with a casting thickness above 400 µm exhibited macrovoids, whereas denser, sponge-like structures were found in thinner-cast membranes. It was speculated that more interaction between the polymer and solvent occurred in thicker membranes, resulting in incomplete solvent diffusion and subsequently reduced shrinkage [106]. Similarly, a critical structure-transition thickness of 12 µm was found P84 (BTDA-TDI/MDI co-polyimide)/NMP membranes where the morphology transitioned from a sponge-like structure to finger-like structure with increasing thickness [107].

The scale of the membrane casting, that is, doctor blade extrusion at the small batch scale and slot die casting on a roll to roll (R2R) at the large/continuous scale, have also been observed to affect the morphology of membranes [108,109]. Membrane fabrication at laboratory scale is performed with a casting knife without any set velocity using a stationary substrate. The dope solution is poured on the substrate manually and is spread over the substrate without pre-determined flow rate and velocity by hand. Conversely, slot die casting on a R2R involves predetermined process parameters, such as flow rate of the polymer dope and velocity, hence, the dope is subjected to shear. Therefore, the chosen fabrication method can cause differences in the membrane structure.

## 3. Measures of System Compatibility

In order to introduce a new solvent mixture into the NIPS fabrication process, several factors need to be taken into consideration, namely the Hansen solubility parameter model, viscosity of the dope solution, ternary phase diagram of the polymer/solvent/nonsolvent system, and diffusion rate of the solvent and non-solvent [109,110,111]. Together, these factors contribute to modelling the thermodynamic and kinetic aspects of the system, as well as a clearer understanding of the phase inversion process.

### 3.1. Hansen Solubility Parameter

The interactions between the components of a dope solution (e.g., polymer, solvent, non-solvent) can influence the polymer behavior in the solution and the progression of phase inversion and mutual solubility parameters can be used to determine these interactions. In particular, the Hansen solubility parameters account for the dispersion forces, polar forces, and hydrogen bonding to calculate three partial solubility parameters [112]. The affinity of the polymer and solvent, deemed *R_a_*, can be calculated using Equation (1), as shown:

(1) $$R_{a}=\sqrt{4{\left(\delta_{d2}-\delta_{d1}\right)}^{2}+{\left(\delta_{p2}-\delta_{p1}\right)}^{2}+{\left(\delta_{h2}-\delta_{h1}\right)}^{2}}$$

where *δ_d_* represents energy density from dispersion bonds, *δ_p_* is energy from the dipolar intermolecular force, and *δ_h_* is energy from hydrogen bonds. A small *R_a_* value indicates favorable compatibility of the polymer and solvent [103]. As another component of Hansen solubility parameter theory, the parameters of a polymer and solvent form a sphere. The relative energy difference (*RED*) can describe the interaction between the polymer and solvent and can be calculated using Equation (2):

(2) $$RED=\frac{R_{a}}{R_{0}}$$

where *R_0_* represents the radius of the Hansen solubility parameter sphere for the polymer. A *RED* value equal to or less than 1 indicates a suitable solvent for the polymer [103].

### 3.2. Viscosity of the Dope Solution

Akin to the Hansen solubility parameter, the viscosity of the dope solution relates to the hydrogen bonds between the polymer and solvent. As such, the viscosity can be measured to monitor the mixing process of the solution and estimate the optimal mixing time and temperature. During mixing, the viscosity increases as dissolution progresses, reaching a maximum value once the polymer is fully dissolved in the solvent, thus, reaching an equilibrium state. The viscosity change over shear rate can be measured to identify the liquid behavior of dope solution under shear force. By enabling the estimate of the viscosity at any point of time during the casting process, the relationship with the viscosity of shear rate provides quantified support in membrane casting and can subsequently be used to manage the flow behavior [104].

### 3.3. Ternary Phase Diagram

**Figure 9:**
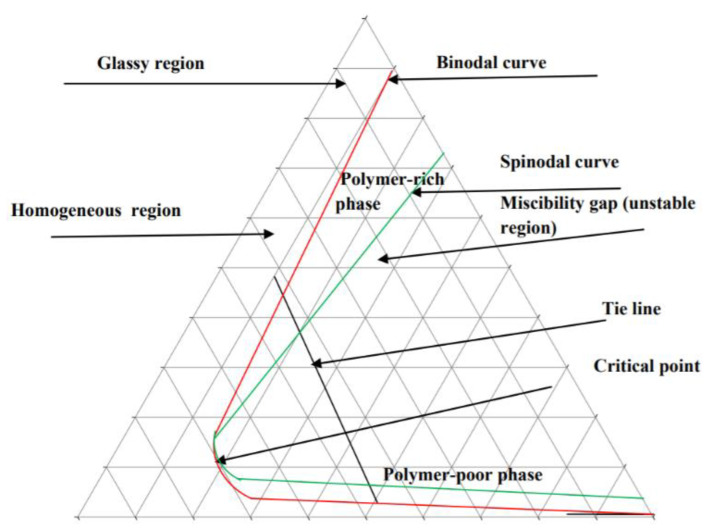
Ternary phase diagram of polymer-solvent-non-solvent mixture. Reprinted from [113], Copyright 2012 Australian Journal of Basic and Applied Sciences (AJBAS).

Once a polymer/solvent/non-solvent mixture is selected, a ternary phase diagram can be generated to predict the possible de-mixing behaviors of the dope solution. Each corner of the diagram represents a pure component; the boundary lines between corners represent two-component mixtures, while the space inside the diagram indicates the presence of all three components. The features found within the phase diagram, including the spinodal and binodal curves, critical point, and tie lines, contribute to characterizing the phase behavior of the mixture [113]. The ternary phase diagram, illustrated in Figure 9, displays these aspects for a polymer-solvent-non-solvent mixture. The ternary phase diagram theoretical curves are significant when a new polymer or solvent is to be investigated to fabricate membranes because these curves can quantitatively guide the specific polymeric membrane formation, including the compositions of the polymer/solvent/nonsolvent system and the prediction of morphology of the membranes.

Constructing a ternary phase diagram can be done by defining the thermodynamics of the system. Namely, cloud point measurements obtained via titration can be organized into a cloud point curve; for a ternary system, the cloud point curve serves as the binodal curve. Determining the spinodal curve involves extrapolation of the isothermal compressibility, heat capacity, or diffusion coefficient of the system as it transitions from a stable to metastable state [104].

A ternary phase diagram of polymer/solvent/water, as shown in Figure 9 is also commonly used to characterize the de-mixing processes in the phase inversion process. In Figure 9, the binodal curve is the liquid-liquid phase boundary, and the line that connects the two points of equilibrium compositions is the tie line. Any composition inside the binodal curve de-mixes into two different composition points, polymer-rich and polymer lean phases, which are in thermodynamic equilibrium. The composition points outside the binodal curve are in the same liquid phase. For the instantaneous de-mixing process, when the liquid film immerses into water, the liquid film de-mixes immediately into a polymer-rich phase and a polymer-lean phase. For the delayed de-mixing process, following immersion into water, the liquid film remains outside the binodal curve, thus, indicating that the delayed de-mixing process was a relatively slow process.

**Figure 10:**
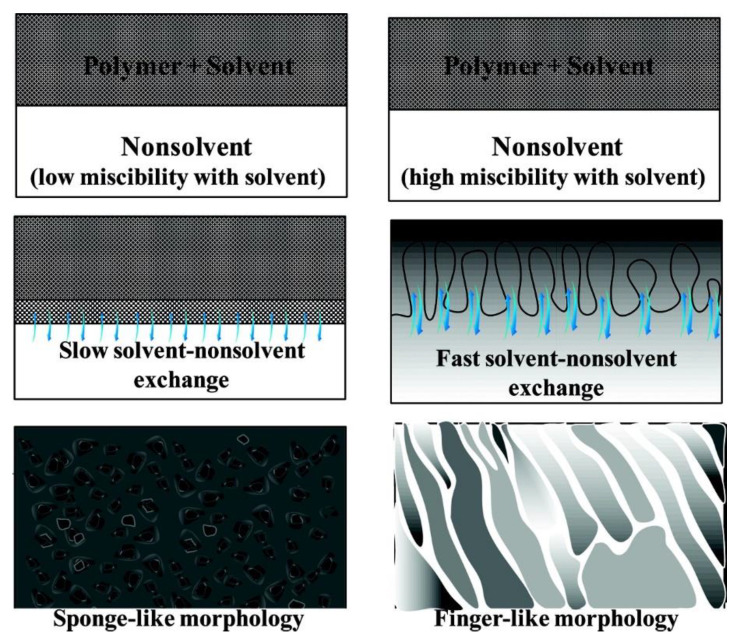
Cross sectional morphologies of membranes formed by instantaneous and delayed de-mixing processes. Reprinted with permission from [16]. Copyright 2011 American Chemical Society.

Different de-mixing processes may be due to different factors; for instance, the miscibility of solvent in non-solvent and the viscosity of the polymer/solvent liquid film [110,114,115,116]. Low miscibility of solvent in non-solvent leads to a delayed de-mixing process, while high miscibility of solvent in non-solvent results in an instantaneous de-mixing process [114,115]. Similarly, high viscosity of the dope solution may lead to a delayed de-mixing process, and low viscosity may lead to an instantaneous de-mixing process [110,116]. As shown in Figure 10, for an instantaneous de-mixing process, the solvent/non-solvent exchange is fast, and finger-like structures form; for a delayed de-mixing process, the solvent/non-solvent exchange is slow, which results in spongey-like structures.

### 3.4. Diffusion Rate of Solvent and Non-Solvent

Aside from the thermodynamic aspects of mixture compatibility, one of the main kinetic aspects is the diffusion rate of solvent out of the dope solution and non-solvent into the solution, which can be used to quantify the de-mixing process. During the phase inversion process, the chemical potential gradient is highest between the polymer solution and non-solvent, as well as the exchange rate of solvent and non-solvent; over time, the diffusion rate decreases as the concentration gradient levels out [104].

### 3.5. Case Study

Dong et al. [111] calculated the Hansen Solubility Parameters of PolarClean, GVL and their mixtures and theoretically determined that they are suitable to dissolve polysulfone resin. Then, a ternary phase diagram was developed using cloud point titration method to thermodynamically predict the de-mixing behaviors of polymer/solvent system. Viscosity of the dope solutions was then measured to determine their liquid behavior during casting and guide further formulation. Last, during phase inversion, the diffusion rate of solvent/non-solvent was measured to predict the cross-section morphology of membranes from a kinetic perspective. These four measures were employed to study the system compatibility and could be used as a protocol for further study.

## 4. Evaluation of Membrane Sustainability

One of the main concerns for the use of green polymers and solvents in membranes is the sustainability of polymer and solvent production. While a membrane comprised of green components may minimize direct environmental impacts, the use of polymer/solvent manufacturing processes that produce significant environmental impacts would offset the benefits of the product. As such, an evaluation of the environmental and health impacts of these components with a scope spanning from raw material extraction to end-of-use should be considered.

**Figure 11:**
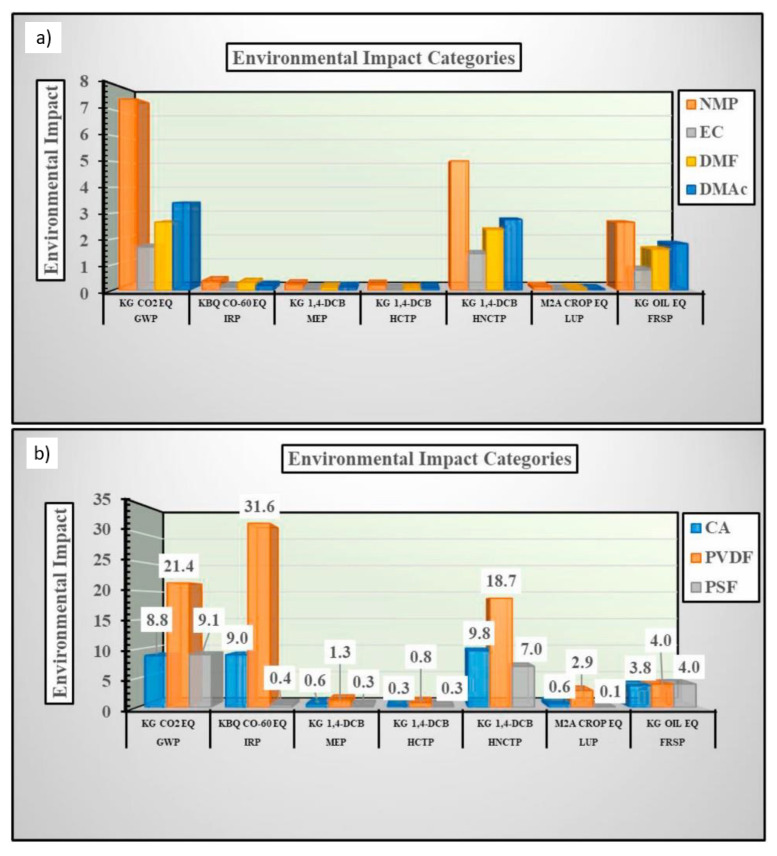
Magnitude of potential environmental impacts for producing 1 kg of conventional and green polymers; and (**a**) solvents (**b**). Reprinted from [117], Copyright 2021 Elsevier.

Life Cycle Assessments (LCA) are a common method for quantifying impacts and can be applied to evaluating membrane sustainability. The main objectives of an LCA are to identify stages in the life cycle of a product that significantly contribute to environmental impacts and to determine how a process influences alter the environmental impacts [117]. Several studies have utilized the LCA framework to evaluate membrane sustainability. Yadav et al. [117] performed an LCA to determine the changes in potential environmental impact for substituting traditional solvents (e.g., NMP, DMAc, DMF) with ethylene carbonate (EC) as a green solvent, as well as substituting PSf and PVDF with CA. Potential impact categories, used for LCA, included global warming potential (GWP; kg CO_2_ eq.), ionizing radiation potential (IRP; kBq Co-60 eq.), marine ecotoxicity potential (MEP; kg 1,4-DCB), human non-carcinogenic toxicity potential (HNCTP; kg 1,4-DCB eq.), land use potential (LUP; m^2^a crop eq.), and fossil resource scarcity (FRSP; kg oil eq.) [117]. The magnitude of each impact category (scaled from 0 to 30) for producing 1 kg of poly mer and solvent are displayed in Figure 11.

Based on the evaluation, the substitution of PSf and PVDF with CA produced mainly minor reductions in environmental impacts. One likely reason is that converting cellulose into CA to improve its solubility involves the use of chemicals and the generation of byproducts that contribute to environmental impacts. However, the integration of green acetylation and bio-derived feedstocks in CA production would mitigate these impacts [117].

To evaluate the environmental impacts of green solvent use, a PVDF/EC system was compared to traditional solvent systems of PVDF/NMP, PVDF/DMAc, and PVDF/DMF. It was determined that EC accounted for only a minor portion of the environmental impacts of the system, whereas NMP, DMAc, and DMF produced larger contributions to the system. Additionally, a lower magnitude of impact was measured for the production of 1 kg of EC in comparison to the traditional solvents (illustrated in Figure 9) [117].

However, the use of LCAs to measure membrane sustainability remains limited, as is the scope of existing LCAs on membrane sustainability. To gain a more thorough evaluation of the integration of green components into membranes, future LCA should examine the impacts related to membrane maintenance and disposal/recycling, including a wider range of green polymers and solvents, and expand to take a Life Cycle Thinking (LCT) approach that considers the social and economic aspects [118].

## 5. Scaling Up the Fabrication Process Using Green Solvents

### 5.1. Scale Up of the Membrane Fabrication Process

Despite extensive research on membrane development and fabrication at the small, laboratory scale [22,75,86,96,102,119], there is a dearth of studies and reports on scaling up membranes. There is substantial research activity in laboratories on casting polymeric membranes. However, many of these methods, such as doctor blade casting, spin coating, dip coating, etc., only work in a batch mode and cannot be easily transferred to large-scale roll-to-roll (R2R) methods [109]. Recently, there have been studies on the scale-up of plain membranes using profile roller coating [109] and slot die casting embedded on roll-to-roll (R2R) systems [115,120,121]. Slot die casting is the most prominent method because it is capable of scaling up thin films across a broad array of areas, while retaining the functionality of the films [122,123].

**Figure 12:**
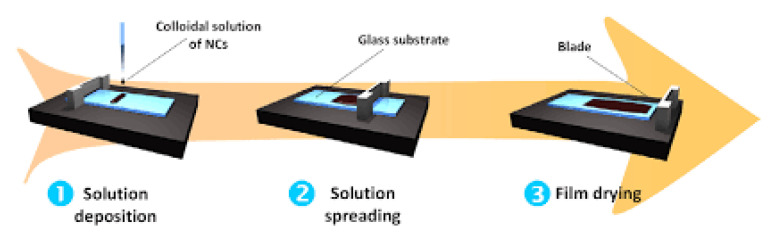
The doctor blade coating process. Reprinted from [125], Copyright 2013 IOP Publishing.

Here, the doctor blade and slot die casting methods are compared as examples of the different fabrication scales. As shown in Figure 12, the doctor blade casting process begins with a dope solution being placed on a substrate, while the doctor blade is positioned at a set height above the substrate. The blade is then moved at a constant velocity to spread the solution onto the substrate to form a film [124].

**Figure 13:**
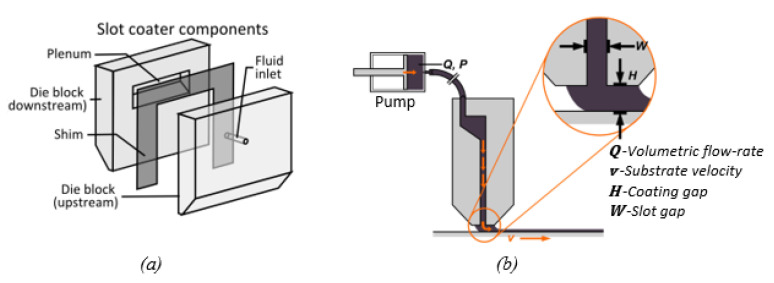
The slot die coating process: (**a**) the slot die head structure; (**b**) the schematic of slot die coating process.

In the slot die casting process, a slot die, as illustrated in Figure 13a,b, is used to deposit a liquid solution onto a substrate that is moving at a constant velocity to form a liquid film on the substrate, as shown in Figure 11b. The difference between the slot die and doctor blade coating methods is that the slot die casting process is pre-metered, and is thus, more flexible in terms of obtaining a wide range of film thicknesses.

### 5.2. Comparison of Doctor Blade Casting and Slot Die Casting of Membranes

Doctor blade casting has been a popular primary film casting method in the laboratory [126], though it is not always the best method to fabricate membranes on an industrial scale because the membrane morphology is largely reliant on the viscosity of the dope solutions and, therefore, not suitable for continuous casting [127,128]. Conversely, slot die casting is a well-developed and commonly used method to manufacture polymer films. It is suitable for continuous casting of liquid films and has been subsequently investigated to scale up polymeric membranes [121,123]. In manufacturing of thin films, there is a constant demand to increase processing speed while maintaining film thickness to fit applications in coating industry. However, several factors, including solution properties, fabrication process and processing parameters can affect the film quality [120,122,127]. Failure to balance critical factors, such as processing speed and flow rate, can lead to defects on resulting films. Scaling up from a small production scale, such as doctor blade to a larger one, such as slot die, is not intuitively obvious and several parameters must first be identified to determine dope solution flow rate and substrate velocity. One such parameter is the surface tension of the polymer since coating speed depends on surface tension values, as solutions with lower surface tension can restrict the coating speeds to lower values [127]. Another key parameter is viscosity, which plays a significant role in determining the processing conditions, specifically the flow behavior and consequently fabrication defects, such as air bubble entrapment, which is reduced in lower viscosity solutions [120,122,127].

### 5.3. Advantage of Slot Die Casting for Scale Up

**Figure 14:**
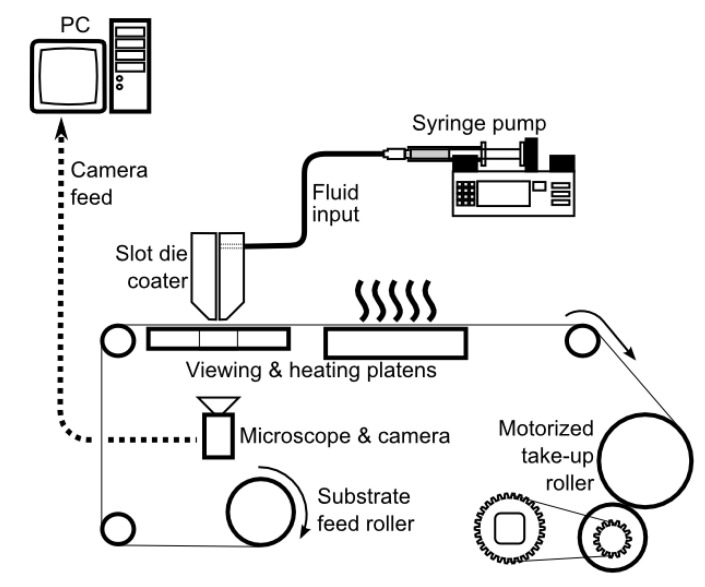
Schematic of a simple R2R experimental setup with a slot die coater. Reprinted with permission from [121]. Copyright 2020 John Wiley & Sons.

In most cases, laboratory methods, such as doctor blade casting, spin coating and solution casting, are not scalable and/or do not introduce the same stress on the membrane as those formed using scalable approaches, e.g., slot die casting. Furthermore, different manufacturing techniques may lead to significantly different membrane properties (e.g., mechanical, chemical, etc.), microstructure, particle distribution, overall membrane functionality, and its ability to treat water. The integration of slot die casting into a R2R system allows for continuous casting of polymeric membranes. An illustration of a simple R2R system embedded with a slot die coater is shown in Figure 14, which has been used to study scale up of microfiltration, ultrafiltration and AgNP composite membranes previously [111,128]. This system allows for pumping a dope solution at a preset flow rate between two die halves onto a moving substrate positioned below the slot die by a small distance.

**Figure 15:**
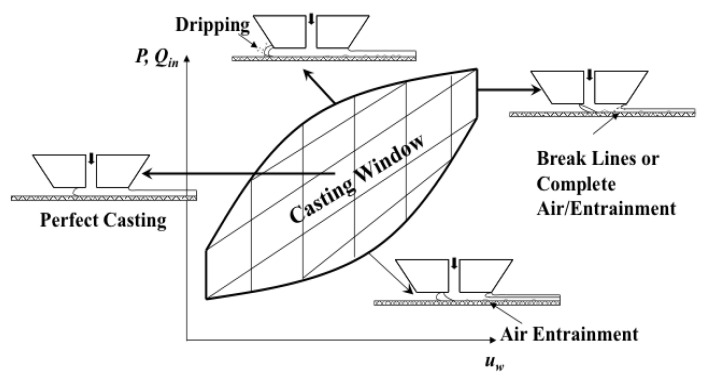
Schematic of a generic casting window to illustrate the upper and lower boundaries of a slot die casting process before the onset of defects. Reprinted with permission from [120,130]. Copyright 2013 Elsevier.

The slot die process offers the advantage of controlled coating thickness, (*h*) as it is only a function of specific process parameters: Pre-metered flow rate of solution per unit die width (*Q’*) and substrate speed (*u_w_*) [127]. That is, *h* = *Q’/u_w_*. Other process parameters, such as the slot gap width (*W*) and the coating gap height (*H*), can affect the quality of the film as cast with defects being introduced into the film if the process is not properly controlled. The space of process parameters in which defect-free casting can occur for a given coating fluid is called the casting/coating window [129]. A graphical representation of this space as a function of the volumetric flow rate and the substrate speed during the coating process and the associated defects seen at the boundaries is shown in Figure 15.

### 5.4. Case Study of Scale Up

Dong et al. [111] used the slot die technique to explore the feasibility to scale up PSf membranes, using PolarClean and GVL and PolarClean/GVL mixtures and compared it with the doctor blade technique and using the traditional solvent DMAc. Viscosity was an important factor in this investigation, as it was used to quantify the mixing time required to dissolve the polymers, using the solvents, and to study the liquid behavior of dope solutions. Adding GVL into PolarClean effectively reduced the mixing time needed to dissolve PSf and decreased the viscosity of the dope solution to enhance the casting window. The membranes prepared using the solvent mixture of 50% GVL and 50% PolarClean and the slot die technique displayed similar operational parameters, including flux decline, permeability and filtration performance, compared to the membranes prepared using DMAc and the doctor blade technique.

In view of climate change and the increasing scarcity and rising prices of natural resources, improving resource efficiency is becoming an increasingly significant factor in manufacturing sector. Eco-manufacturing is essential for achieving sustainable green growth, as well as being economically sensible. In eco-manufacturing, no wastes should be produced, and any by-products should be broken down by microbial action to produce non-hazardous products. Eco-friendly products are also made as little as possible from harmful chemicals and toxic compounds, such as petroleum-derived polymers and solvents, wood preservatives or creosote, volatile organic compounds, chlorine, among others. The products should also serve multiple purposes. An example of an eco-friendly product is a cloth bag made from jute or hemp that can be used for numerous tasks, such as bagging groceries and carrying books, and it lasts for years. Eco-manufacturing is an emerging new best practice in manufacturing and other industries. Eco-friendly machinery and installation of greener machinery parts would also help to reduce the overall carbon footprint.

## 6. Conclusions and Perspectives

Given the hazards of traditional solvents, there is an urgent need to identify alternative, green solvents for polymeric membrane fabrications that are inherently safer and meet regulations. In addition, the use of green polymers in membranes would further reduce the environmental impacts. As such, there are numerous studies on the use of different eco-friendly polymers and solvents in polymeric membrane fabrication. However, future studies should further evaluate the sustainability of green component manufacturing and integration with membranes.

While a plethora of eco-friendly solvents have been studied to cast polymeric membranes, the scale-up study has not been heavily addressed. More casting techniques need to be investigated in order to introduce novel green solvents into the membrane fabrication process at an industrial scale. Aside from the regulatory pressure of banning current petroleum-derived solvents, two other factors can contribute to developing the use of green solvents in industry. First, the proposed green solvents can be directly used on the current casting manufacturing line, thus, no, or minimum, capital investment is required. Secondly, affordable solvents with a reliable supply chain would increase the possibility for green solvents being considered in industry. Considering all factors, there is no doubt that eco-friendly solvents will be used in membrane manufacturing processes as promising solutions continue to emerge.

## Figures and Tables

**Table 1:** Comparison of four phase separation methods [30].

	NIPS [16]	TIPS [31]	VIPS [29,32]	EIPS [27]
Principle	Mass Transfer	Heat Transfer	Mass Transfer	Mass Transfer
Components	Polymer	Polymer	Polymer	Polymer
	Solvent	Solvent	Solvent	Solvent
	Non-solvent		Non-solvent (vapor)	Non-solvent
Advantages	Diverse porous structure, high selectivity, low operation temperature	Easy control, uniform structure, good reproducibility	Crystallization, gentle formation process	Good reproducibility
Disadvantages	Many operation parameters, finger-like pore structures do not have good mechanical strength	High energy consumption, requirements for solvents: low molecular weight, high boiling point, low volatility, high miscibility with polymers, thermal stability	Many operation parameters, energy consumption	Difficult to find suitable solvents and nonsolvents used in EIPS

## Data Availability

The data presented in this study is based on a literature review of published materials and are available on request from the corresponding author.
